# Towards an efficient compression of 3D coordinates of macromolecular structures

**DOI:** 10.1371/journal.pone.0174846

**Published:** 2017-03-31

**Authors:** Yana Valasatava, Anthony R. Bradley, Alexander S. Rose, Jose M. Duarte, Andreas Prlić, Peter W. Rose

**Affiliations:** 1 Structural Bioinformatics Laboratory, San Diego Supercomputer Center, University of California, San Diego, La Jolla, CA, United States of America; 2 RCSB Protein Data Bank, San Diego Supercomputer Center, University of California, San Diego, La Jolla, CA, United States of America; Wake Forest University, UNITED STATES

## Abstract

The size and complexity of 3D macromolecular structures available in the Protein Data Bank is constantly growing. Current tools and file formats have reached limits of scalability. New compression approaches are required to support the visualization of large molecular complexes and enable new and scalable means for data analysis. We evaluated a series of compression techniques for coordinates of 3D macromolecular structures and identified the best performing approaches. By balancing compression efficiency in terms of the decompression speed and compression ratio, and code complexity, our results provide the foundation for a novel standard to represent macromolecular coordinates in a compact and useful file format.

## Introduction

The Protein Data Bank (PDB) [1], the archive for 3D structures of biological macromolecules, has rapidly grown over the last few years. Developments in the major experimental techniques enable high-throughput structure determination and the number of deposited structures now exceeds 124,000 entries, increasing by about 10,000 entries per year. The PDB is not only growing in numbers, but newly released PDB entries are also growing in complexity. New integrative methods that combine multiple modelling and experimental techniques, most notably Electron Microscopy, now determine structures of up to the megadalton (MDa) range at atomic resolution [2–4].

Such large complexes bring major challenges to analysis and visualization tools since transfer and processing of the structural data is slow. Limitations in network bandwidth and client side memory further reduce the visualization performance on the web and mobile devices. These bottlenecks are caused by inefficiencies of the file formats currently used by the PDB to store macromolecular structures. The PDBx/mmCIF file format [5], the archival text-based format for the PDB, is flexible, extensible and verbose with rich metadata that can represent structures of any size but is not optimized for fast loading and parsing of structural data. The legacy PDB format [6] is a less verbose and more compact textual format, but only supports structures with less than 100,000 atoms. However, the largest structure in the PDB, the HIV-1 virus capsid (PDB ID: 3J3Q) [7], contains more than 2.4 million atoms and takes up 254 MB of disk space when stored as an uncompressed PDBx/mmCIF file. As we expect the deposition of even bigger structures in the future, the development of a novel compact representation of macromolecular structures is necessary. Our goal is a companion format to the PDBx/mmCIF archival format that is designed for the needs of structural data visualization, analysis and transmission over the Internet. Given limited network bandwidth, the reduction in file size is necessary for efficient data transmission. Hence we propose to store the structural data in the encoded and compressed form. The goal of this paper is to evaluate strategies to compress macromolecular coordinates, since a large fraction of the data in structure files are the atomic coordinates. The encoding and compression strategies described here form the basis for the MacroMolecular Transmission Format (http://mmtf.rcsb.org), a file format for the efficient transmission of structural data for interactive visualization and analysis applications, especially for large molecular complexes.

Macromolecules have structural redundancy: they have repeated or similar structural elements and predictable local geometry. Hence we propose to compress macromolecular coordinates employing strategies that consider bespoke structural features. This offers an opportunity for more efficient compression than general-purpose compression algorithms, such as GZIP.

A previous effort to compress the coordinates of individual PDB structures was made by [8]. In their approach, size reduction was achieved by reducing the precision of coordinates to two decimal places, making it a lossy strategy. Other efforts in macromolecule coordinate compression have appeared within the context of the molecular dynamics field, where simulations can produce terabytes of coordinate trajectories. The applied methods there have achieved high compression ratios using various inter-frame encoding schemes, e.g., delta coding, prediction with polynomials, or space curves [9–11]. As these methods focus on compressing coordinate trajectories they are of limited value for compressing coordinates of individual structures in the PDB archive.

In the following, we systematically investigate compression strategies applied to the 3D atomic coordinates of macromolecules that target the structural redundancy and spatial adjacency rather than syntactic redundancy. We explore lossy as well as lossless compression approaches, investigate intramolecular as well as intermolecular compression achieved using different encoding strategies. Each of the compression methods is evaluated based on key performance metrics. The results provide a strategy for compressing macromolecular coordinates that balances compression efficiency and implementation complexity.

## Materials and methods

In this article, we focus on the compression of 3D coordinates of macromolecules as they are challenging for general purpose compression techniques. General compression tools such as GZIP are efficient when the redundancy in data is high, like the redundancy of the language in a text (e.g. repetitive words). For example, GZIP locates repetitive strings within a text file and replaces those strings temporarily with shorter codes to make the overall file size smaller. However, the coordinates coming from experimental data generally do not exhibit such syntactic redundancy. The proposed approaches use the knowledge about structural features of biological macromolecules to create a compact representation of their atomic coordinates. Specifically, we developed two types of strategies: (i) intramolecular compression that operates on the sequence of atoms within a polymer chain; and (ii) intermolecular compression designed for the compression of special cases of multiple chains with identical atoms, such as NMR models and structures with repeated identical subunits.

### Intramolecular compression

Intramolecular compression operates on the coordinates of individual polymer chains. This method exploits spatial adjacency and connectivity of atoms consecutively linked to form a polymer chain. The dataset used to test intramolecular compression strategies consist of the three-dimensional structures present in the PDB (http://www.pdb.org) as of August 2, 2016. The total number of structures was 121,407 with a total size of the gzipped mmCIF files of 29.7 GB.

### Intermolecular compression

Intermolecular compression methods operate on identical molecules, i.e., those that contain the same number of identical atoms: (i) ensembles with multiple models; (ii) structures with identical subunits related by non-crystallographic symmetry; (iii) asymmetric structures with repeated identical subunits (e.g., the HIV capsid). In contrast to intramolecular strategies, intermolecular approaches compare corresponding identical atoms across molecules instead of consecutive atoms within each molecule. The order in which the molecules are compared is defined by the *traversal strategy*. The general idea is that the information can be stored for a single molecule and all other molecules can be referenced to a representative molecule.

#### Ensembles with multiple models

The first dataset we used to evaluate intermolecular compression included 8108 NMR structures and 250 X-ray structures that contain multiple models. Such models reflect the dynamic nature of biopolymers. They represent a single structure as an ensemble of conformations that satisfy experimental restraints.

**Fig 1:**
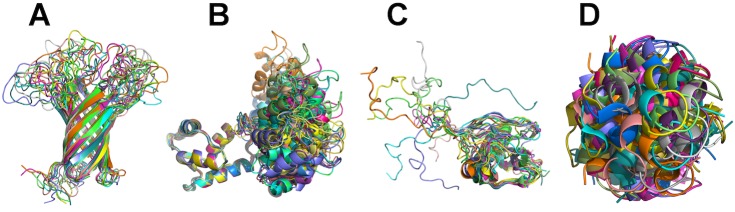
Examples of NMR structures with multiple models. (A) “well-defined” structure (PDB ID: 1G90); (B) rigid domains connected with flexible linker (PDB ID: 1CFC); (C) lacking a consensus structure (PDB ID: 2MW5); (D) unaligned models (PDB ID: 1QP6).

Structural ensembles have varying degrees of flexibility (Fig 1) and structures are typically aligned by the authors before the deposition. In few cases, the structures are not aligned (Fig 1D). The total number of structures with multiple homogeneous models is 8,358, which accounts for 6.9% of the total number of structures in the PDB archive. In terms of size, this dataset occupies 4.6 GB as GZIP compressed mmCIF files, which constitutes 15.5% of the PDB archive size.

#### Structures with identical subunits related by non-crystallographic symmetry

**Fig 2:**
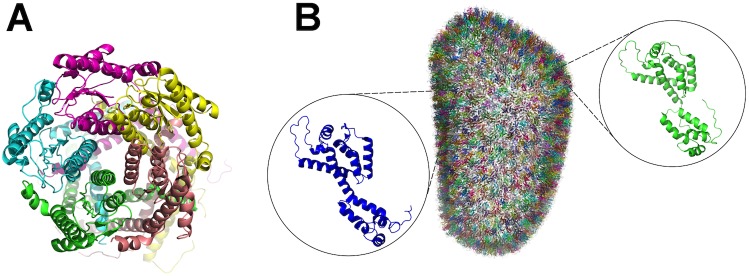
Examples from the dataset of structures with identical subunits. (A) non-crystallographic symmetry between identical subunits with similar conformations (PDB ID: 4Y7J) and (B) repeating identical subunits with similar conformations (PDB ID: 3J3Q).

The second dataset we used to test intermolecular compression includes oligomeric complexes composed of identical protein subunits. Those subunits have the same amino acid composition and very similar 3D structure (Fig 2). The proteins with identical subunits account for 45,477 structures (37.5% of total number of structures in the PDB archive), of which 15,179 structures are homogeneous (15.5% of a total number of structures in the PDB archive), by which we mean that identical subunits have the same number of atoms.

#### Traversal strategies

**Fig 3:**
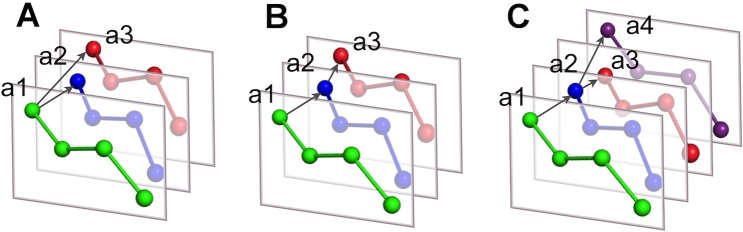
Traversal strategies. The traversal strategies to define the encoding order of molecules for the intermolecular compression methods: (A) reference first: atoms from molecules 2 (a2) and 3 (a3) are referenced to an equivalent atom from molecule 1 (a1); (B) waterfall: the order to compare molecules is sequential (a1-a2-a3); (C) the order is defined by the Minimum Spanning Tree build for the set of molecules.

Unlike intramolecular compression methods that follow the linear order of atoms to encode their coordinates, for the intermolecular compression methods there are N! permutations to compare N coordinate sets. We have implemented three traversal strategies that define the order in which the molecules are compared (a molecule can be an entire model in a multi-model structure or a single subunit). These strategies include: (i) *reference first*, e.g., the first molecule is used as a reference for every other molecule (Fig 3A); (ii) *waterfall*, e.g., the molecules are traversed subsequently, such as the second molecule goes after the first, the third after the second and so on (Fig 3B); (iii) *Minimum Spanning Tree* (MST), e.g., the molecules are represented as an undirected connected weighted graph, from which an MST is built (Fig 3C).

Each vertex of the MST graph is a molecule and vertices are connected by weighted edges. The weight between two connected vertices results from comparison of two molecules. We implemented two edge weight metrics:

1. RMSD—the average distance between the identical atoms of two molecules calculated as follows:
  $$RMSD=\sqrt{\frac{\sum_{i=1}^{N}{{\Delta x}_{i}}^{2}+{{\Delta y}_{i}}^{2}+{{\Delta z}_{i}}^{2}}{N}}$$ (1)
  where ${\Delta x}_{i}=x_{i}^{2}-x_{i}^{1}$, ${\Delta y}_{i}=y_{i}^{2}-y_{i}^{1}$, ${\Delta z}_{i}=z_{i}^{2}-z_{i}^{1}$, and *N* is the number of atoms.
2. GZIP—the distances between the coordinates of identical atoms of two molecules are calculated and the result is compressed using the GZIP algorithm.

We used Prim's algorithm to build the MST [12] implemented in JGraphT package (http://jgrapht.org). The MST is an undirected graph with the least total sum of all weights. To define the traversal order for each graph we calculate the root as the longest path that connects the two most distant vertices, and branches, shorter sequences that originate from the root nodes. The root is found by traversing the MST twice using the breadth-first algorithm. For example, the traversal order for four molecules can be as shown on Fig 3C, the root connects 1^st^, 2^nd^ and 3^rd^ molecules and the branch connects 2^nd^ and 4^th^ molecules.

#### Superposition to improve intermolecular encoding

For intermolecular compression methods, we superpose each pair of molecules before encoding. The superposition is useful for multi-model structures if models are not aligned by the authors. For the structures with identical subunits superposition is required to make the intramolecular encoding beneficial. We used a *least squares fit* algorithm, a conventional superposition method that minimizes the sum of squared residuals between atomic coordinates.

To restore the original coordinates the transformation must be stored for each superposition. Each transformation requires 24 additional bytes to store the translation and 32 bytes to store a rotation in form of quaternion. This number is much smaller than the size of coordinates and was not included in the calculation of the compressed size.

### Full versus reduced representation of PDB structures

We analyzed the performance of compression algorithms on the PDB structures in two representations named full (F) and reduced (R). The most detailed full representation includes all atoms from the macromolecular structure that belong to the polypeptide or polynucleotide chains. However, the level of detail in full representation is not always necessary. The reduced representation considers only positions of alpha-carbon atoms for polypeptide chains and central phosphorus atoms of phosphate groups for polynucleotide chains. Using a lightweight reduced representation speeds up file transfer and rendering significantly, providing enough information for analysis. One example of a reduced representation application is the visualization of large macromolecules, where the visualization of surfaces or ribbon diagrams is often preferred as an atomic representation becomes overcrowded. The reduced representation has also been shown to be useful for computational comparison of two protein structures, structure prediction, and structure modeling [13,14].

### Lossless versus lossy compression

**Fig 4:**
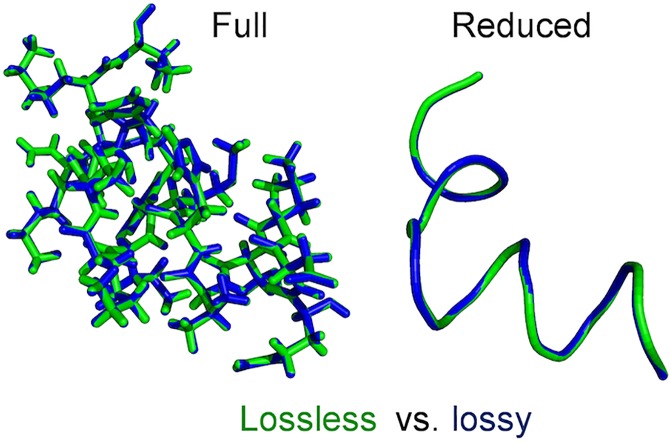
Lossless vs lossy representation of a protein structure. This example shows that the difference between lossless and lossy representations (0.1 Å precision) of a protein structure is at the visual perception limit.

Data compression generally comes in two flavors, lossy (LS) and lossless (LL). Lossless compression restores the original data perfectly (bitwise identical) from the compressed data, in contrast to lossy compression where the full precision of the original data is irreversibly lost after compression, but higher compression ratios can be obtained. We achieve lossy compression by reducing the precision of coordinates to one decimal point, i.e., 0.1 Å. In the visualization of macromolecular structures the precision of 3D coordinates can be sacrificed if lossless and lossy representations look very similar. Visual inspection shows that a lossy representation still maintains reasonably “ideal” geometries for both full and reduced representations (Fig 4).

### General compression scheme

**Fig 5:**
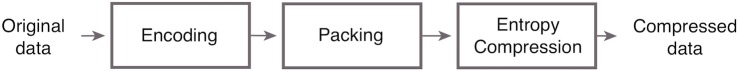
Data flow through compression algorithms. Original data are 3D coordinates of macromolecular structure. At the encoding step the coordinates are transformed to smaller values using one of the encoding algorithms described in this section. Packing converts integer values to a sequence of bytes optimizing the number of bits used to store each value. Entropy compression reduces the syntactic redundancy in encoded data representing by shorter codes symbols that occur more frequently.

The general compression scheme refers to two algorithms: a compression algorithm that takes as an input the original data sequence *S*_*o*_ and reduces it to *S*_*c*_ that requires fewer bits, and a decompression algorithm that recovers *S*_*o*_ from *S*_*c*_. As a general scheme, our compression approaches are based on the following subsequent steps: (i) the *encoding* step, coordinates are transformed from floating point numbers to a more compact integer representation; (ii) at the *packing* step 32-bit integers are encoded as 16-bit integers; (iii) *entropy compression* removes syntactic redundancy in the encoded data (Fig 5). Below we describe algorithms associated with each step.

#### Encoding

Encoding refers to the transformation of atomic coordinates to a representation better suited for compression. Here the encodings are fixed-width, e.g., this representation uses the same number of bits to store the encoded value as to store the original value. The transformation aims to reduce the dynamic range of values, so a smaller number of bits can be used to store the values. In conjunction with the packing and entropy compression methods, described later in the article, such representation yields a higher compression rate. The following encoding strategies were considered for this study.

**Integer encoding:** Macromolecular coordinates are captured as real numbers in Ångstrom (Å, 0.1nm) with a limit on precision, i.e., to within 0.001 Å. Thus, we can represent the atomic coordinates as integers without loss of accuracy by multiplying the coordinate values by a factor of 1000. We also used a smaller multiplication factor of 10 to achieve lossy compression. Using a smaller multiplication factor introduces a loss of accuracy of the original coordinates. However, loss of coordinate accuracy, does not necessarily lead to the loss of experimental data. Experimental measurements determine the atomic position with a degree of uncertainty. The experimental accuracy of the macromolecular coordinates is usually much lower than 3 decimal places. For crystallographic structures the B-factor (in units of Å^2^) describes indeterminable thermal noise and is related to the mean square displacement of the atomic position. In proteins, B-factors typically range from 5 to 60 Å^2^, corresponding to a positional uncertainty of greater than 0.2 Å^2^ [15,16]. NMR structure ensembles do not provide a statistically meaningful description of the true accuracy of coordinates given the experimental uncertainties in deriving distance restraints [17]. Other methods such as EM produce lower resolution structures than X-ray crystallography. This allows us to exploit lossy compression that stores the coordinates up to a tenth of an Å (multiplication factor of 10), which is generally sufficient to preserve the essential structural information provided by lossless representation.

**Delta encoding:** Delta encoding stores the differences between coordinates instead of absolute values. As the atoms in macromolecular structures are within a certain distance range determined by the length of their chemical bonds and spatial adjacency of amino acids, the distance between two consecutive atoms will be typically smaller than the absolute values of their coordinates. For example, a typical carbon-carbon (C-C) covalent bond has a bond length of 1.54 Å [18].

**Fig 6:**
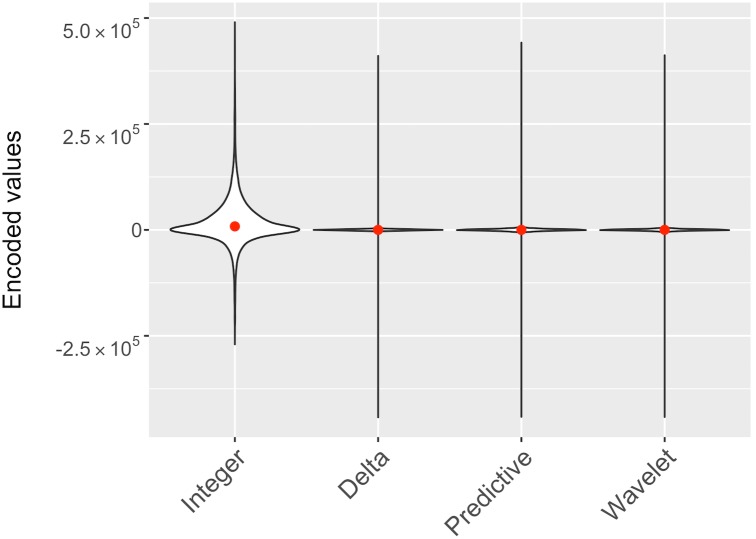
The effect of *Integer*, *Delta*, *Predictive*, and *Wavelet* intramolecular encodings on coordinate values. Violin plot comparing the effect of encoding strategies on the coordinate values. The numbers of structures included in the plot were 1227 randomly selected PDB structures (1% of the PDB archive). The red dot indicates the median value for each set of encoded values. The white region is a kernel density plot representing the distribution of encoded values. The kernel density shows the shape of the data distribution. The wider section of the violin plot represents a higher probability that members of the set will take on the given value; the skinnier section represents a lower probability.

First, the coordinates are encoded as integers are stored in a single array *C* = {*x*_0_ … *x*_*n*_, *y*_0_ … *y*_*n*_, *z*_0_ … *z*_*n*_}, where n is a total number of atoms in the structure. The algorithm stores the first encoded value as *s*_0_ = *c*_0_, all the consecutive values are encoded as *s*_*i*_ = *c*_*i*_ − *c*_*i*−1_, where *c*_*i*_ ∈ C. We can visualize the effect of encoding by plotting the kernel density for the encoded values that shows the shape of the data distribution (Fig 6). After encoding, there is a higher probability of smaller values centered around zero.

**Predictive encoding:** Similar to delta encoding, the predictive encoding works with the sequence of integer encoded coordinates *C* = {*x*_0_ … *x*_*n*_, *y*_0_ … *y*_*n*_, *z*_0_ … *z*_*n*_}, where n is a total number of atoms in the structure. The history of points is used to predict the position of the next point and the errors between predicted and original values are stored. Here, the position of the next atom is predicted based on the distance between preceding pair of atoms. The algorithm stores the first encoded value as an original coordinate, e.g. *s*_0_ = *c*_0_. The second encoded value is *s*_1_ = *c*_1_ − *c*_0_. Then at each step the algorithm calculates and stores the error *e*_*i*_ = *c*_*i*_ − *p*_*i*_, where *p*_*i*_ is a predicted value calculated as *p*_*i*_ = 2*c*_*i*−1_ − *c*_*i*−2_. In this form, this coincides with delta-delta encoding. The effect of the encoding is shown in Fig 6.

**Wavelet-based encoding:** Discrete Wavelets Transforms have made their way into compression as an efficient image compression technique. For example, the biorthogonal CDF 5/3 wavelet transform, also called Le Gall 5/3 wavelet, which performs an integer-to-integer wavelet transform, is used by the JPEG2000 format for lossless image compression [19]. Here we applied this algorithm for lossless compression of macromolecular coordinates. Wavelet encoding uses the wavelet function to transform the sequence of coordinate values into the sequence of wavelet coefficients. The effect of encoding is shown in Fig 6.

**Fig 7:**
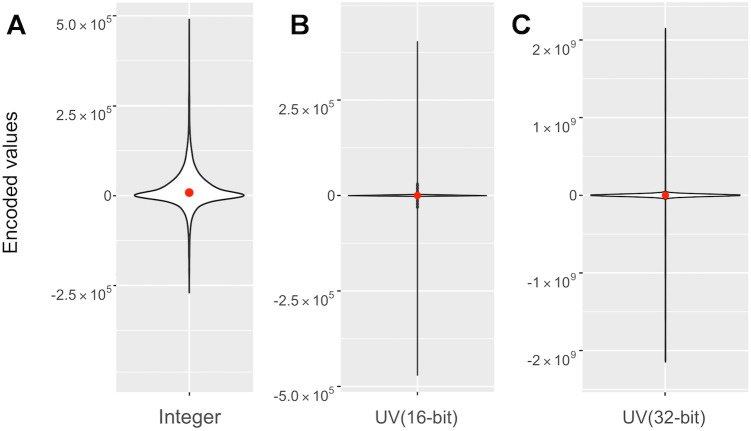
The effect of the unit vector encoding on the coordinate values. Violin plot comparing the effect of unit vector encoding strategies on the integer encoded coordinate values (A), to 16-bit encoding (B), and 32-bit encoding (C). The numbers of structures included in the plot were 1227 randomly selected PDB structures (1% of the PDB archive). The red dot indicates the median value for each set of encoded values. The white region is a kernel density plot representing the distribution of encoded values. The kernel density shows the shape of the data distribution. The wider section of the violin plot represents a higher probability that members of the set will take on the given value; the skinnier section represents a lower probability.

**Encoding based on unit vector compression:** Unit vector compression represents atomic coordinates of a molecule as a set of vectors between every pair of consecutive atoms. The encoding algorithm compresses these vectors as follows: (i) represent each vector by its direction (a unit vector) and length (a scalar value); (ii) compress every unit vector to a single integer value using the unit vector coding technique; (iii) reconstruct the original vector from the decompressed unit vector and original length. In step (i), the length of each vector is subtracted from the average vector length calculated for a given molecule. The average length is stored once and the difference between the actual and average length is stored for every vector. In step (ii), three coordinates of the unit vector 32 bits each (96 bits in total) are compressed to a single 32- or 16-bit signed integer number. The effect of 16-bit and 32-bit encodings is shown in Fig 7. The kernel density for the encoded values shows that higher probability for smaller values with respect to the integer encoded values. However, the values for the compressed unit vector span the entire 16 and 32-bit range of integer values.

The compression algorithm is described in “Compressed Unit Vectors” by David Eberly (https://www.geometrictools.com). Due to the quantization at the step (ii), decompressed vectors contain a rounding error. In the step (iii), we calculate and store the difference between coordinates of original and decompressed vectors to reconstruct the coordinates losslessly.

#### Packing

The packing algorithm allows a compact memory representation of data. As the values of encoded coordinates are within a smaller dynamic range with respect to original values, fewer bits can be used to store and transmit the data. Below we describe the packing strategies that we explored for this work.

**Recursive indexing:** *Recursive indexing* encodes values such that the encoded values lie within the interval between minimum (min) and maximum (max) values [20]. This allows to represent 32-bit integers more efficiently, when most of the values fit into 16-bit (or 8-bit) integers. *Recursive indexing* works as follows: each value that lies within the open interval (min, max) represents itself, otherwise the max (or min if the number is negative) interval endpoint is stored and subtracted from the input value. This process of storing and subtracting is repeated recursively until the remainder lies within the interval.

**Variable-length quantity:** The idea behind the *variable-length quantity* encoding uses variable number of bytes to represent an arbitrary integer. The encoding splits integers into bytes such as 7 bits store the value and 1 bit indicates if the 7 bits of the following byte are part of the original integer (https://rosettacode.org/wiki/Variable-length_quantity#Java).

#### Entropy compression

It is possible to further reduce the size of the encoded and packed coordinates through entropy compression. To meet the requirements of efficient data transmission over the Internet, the method should offer fast decompression and be supported in widely used web browsers. In the following we evaluated the performance of two entropy compression methods that are optimized towards the abovementioned requirements:

GZIP (https://www.ietf.org/rfc/rfc1952.txt) is a lossless compression technique that uses two interplaying algorithms: LZ77, a dictionary-based encoding [21] and Huffman coding [22], which uses fewer bits to encode frequently occurring bytes.

Brotli is a fast compression algorithm developed by Google (http://www.gstatic.com/b/brotlidocs/brotli-2015-09-22.pdf). Similarly to GZIP, Brotli uses the combination of LZ77 and Huffman coding (https://www.ietf.org/rfc/rfc7932.txt).

### Performance metrics

We used different metrics to evaluate the compression efficiency. These include *Shannon entropy* [23], *compression ratio*, and *compressed size*. However, the most objective metric for this study is *compressed size*, i.e. the amount of space required to store the compressed data. We only report the *compressed size* here, since the size of coordinates after encoding and packing may differ for different encoding algorithms.

## Results and discussion

In this paper, we explored various compression methods for macromolecular structures, describing the main ideas behind each technique. We analyzed *intra-* and *intermolecular*, *lossy* and *lossless* compression approaches based on different encoding algorithms. *Lossy* compression can be used in applications that tolerate data loss without noticeable loss of performance, for example molecular visualization. On the other hand, methods such as structure refinement or molecular force field applications may be sensitive to small changes in coordinates. Therefore, *lossless* compression is usually a preferred choice while compressing scientific data and we centered our analysis on the lossless compression algorithms. In the following, we compare the performance of the presented compression approaches and discuss the combination of methods that yield best compression.

### Distribution of encoded values

**Fig 8:**
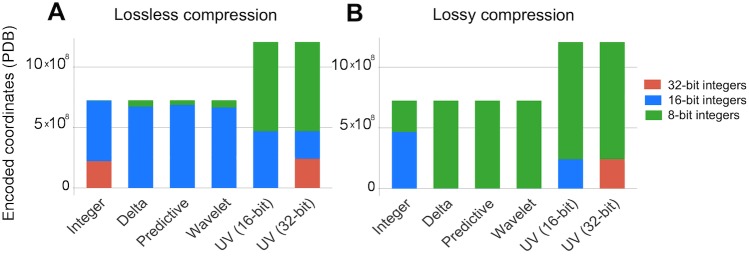
The number of encoded values that can be represented as 32-, 16-, and 8-bit integers in the PDB. Most encoded values can be represented as 16-bit integers for (A) lossless compression and to 8-bit integers for (B) lossy compression for most encoding strategies.

We analyzed the distribution of encoded values for all the structures in the PDB to understand how encoding reduces the amount of space required to store the data (Fig 8). The results indicate that most of encoded values fit into 16-bit integers for lossless and to 8-bit integers for lossy compression. The unit vector (UV) encodings have the following effects: (i) size of encoded data increases by 40% with respect to original size; (ii) 60–80% of encoded values fit into 8-bit integers; (iii) at least 20% of values require 16-bit integers for UV 16-bit (or 32-bit integers for UV 32-bit).

### Packing algorithms performance

The packing algorithms are aimed to create a more compact memory representation of the data. We evaluated the performance of the *recursive indexing* and *variable-length quantity* encodings applied to the results of the intramolecular encoding strategies. *Recursive indexing* outperforms *variable-length quantity* for all encoding strategies except of the UV (32-bit) encoding. For example, the recursive indexing achieves 58% better compression than *variable-length quantity* for the intramolecular delta encoding.

### Entropy compression algorithms performance

We evaluated the performance of Brotli and GZIP compression algorithms by comparing the size of encoded coordinates after compression. Brotli can achieve 3.5% higher compression on average for all encoding algorithms. Though Brotli offers a slightly better compression ratio, GZIP remains a preferred tool for entropy compression due to its wider availability in all browsers and operating systems.

### Intramolecular compression results

In order to obtain a minimum compressed size, we implemented the following intramolecular compression strategies that are built up from a combination of algorithms mentioned above: (i) *Delta* is a combination of integer encoding, delta encoding, recursive indexing, and GZIP compression; (ii) *Predictive* is a combination of integer encoding, followed by predictive encoding, recursive indexing, and GZIP compression; (iii) *Wavelet* runs integer encoding, delta encoding, wavelet encoding, recursive indexing, and GZIP compression; (iv) *UV (16-bit)* is based on the unit vector compression to 16-bits integers, followed by recursive indexing and GZIP compression; (v) *UV (32-bit)* is encoding based on the unit vector compression to 32-bit integer followed by GZIP compression. The recursive indexing step is omitted for UV (32-bit) encoding. We also report the GZIP compressed size of original coordinates when they are represented in memory as *Floating point* numbers and *Integer* encoded values to give a baseline for comparison.

Our results suggest that delta encoding performs best for lossless compression (Table 1), with UV(16-bit) gaining a small advantage over the delta encoding with lossy compression. Delta encoding, however, provides a good trade-off between the compression ratio and code complexity as well as compression/decompression speed. The efficiency of intramolecular delta encoding is mainly due to the regular patterns in spatial adjacency and connectivity of atoms.

**Table 1:** Performance comparison of different intramolecular encodings.

Intramolecular strategies	Size, MB (LL/F)	Size, MB (LS/F)	Size, MB (LL/R)	Size, MB (LS/R)
Floating point	8540.1	8540.1	933.4	933.4
Integer	6778.1	3653.6	723.5	417.3
Delta	**5087.7**	2796.3	**537.5**	326.2
Predictive	5255.1	3082.9	547.1	347.8
Wavelet	5150.5	3003.5	542.9	347.8
UV (16-bit)	5934.2	**2588.9**	659.2	**247.7**
UV (32 bit)	5633.1	4282.9	559.4	436.7

Performance evaluation of intramolecular strategies for lossless (LL) and lossy (LS) compression applied to full (F) and reduced (R) representation benchmarks. This table compares the size of coordinates of all atoms in PDB represented as floating point to the compressed size obtained using intramolecular compression strategies. Recursive Indexing (RI) is applied on top of integer, delta, predictive encodings and encoding using compression of unit vectors into 16-bit integers. The coordinates are subsequently compressed using GZIP.

### Intermolecular compression results

Further we investigated intermolecular compression strategies for the “special cases” of macromolecular structures, such as structures with multiple models and structures with non-crystallographic symmetry or repeated identical subunits. To determine if better compression can be achieved, we compared the results of the proposed intermolecular compression strategies with intramolecular delta compression as we have demonstrated above that the delta compression outperforms other intramolecular compression methods.

We implemented the following tree traversal strategies: reference, waterfall, and Minimum Spanning Tree (MST) and two different metrics to build a weighted graph needed to construct the MST (GZIP, and RMSD). We used delta and predictive algorithms for encoding. To select the best combination of the abovementioned strategies, we broke down the analysis into three steps. First, we evaluated the best metric to construct the MST. Second, we selected the best traversal strategy. Finally, we evaluated the encoding algorithm that yielded better compression. The results of this analysis are summarized in the Table 2.

**Table 2:** Results of intermolecular compression performance using MST traversing strategy with different metrics.

Metric	Size, MB (M/LL)	Size, MB (M/LS)	Size, MB (S/LL)	Size, MB (S/LS)
GZIP size	1014.4	521.3	562.4	**296.4**
RMSD	**1014.3**	521.4	**562.0**	297.6

This table compares the performance of different metrics used to construct the MST. The reported size is a total size of coordinates retrieved from datasets containing the multi-model structures (M) and the structures with repeated subunits (S). Both lossless (LL) and lossy (LS) compression methods were analyzed.

The results suggest no significant difference in compression ratio obtained using different metrics in the construction of the MST. RMSD metric has been chosen for further analysis, which involved comparison of different traversal strategies (Table 3).

**Table 3:** Results for traversing strategies for intermolecular compression.

Traversing strategy	Size, MB (M/LL)	Size, MB (M/LS)	Size, MB (S/LL)	Size, MB (S/LS)
Reference	1013.8	**515.5**	576.0	**295.5**
Waterfall	**1011.4**	520.9	**562.0**	302.8
MST (RMSD)	1014.3	521.4	562.0	297.6

This table shows the comparison of intermolecular traversing strategies. The reported size is a total size of coordinates retrieved from datasets containing the multi-model structures (M) and the structures with repeated subunits (S). Both lossless (LL) and lossy (LS) compression methods were analyzed.

The results indicate slightly better performance for a waterfall strategy, which has been chosen for further evaluation. At the next step the intermolecular delta and predictive encodings were compared with intramolecular delta encoding strategies (Table 4).

**Table 4:** Performance comparison of intermolecular and intramolecular encoding strategies.

Strategies	Size, MB (M/LS)	Size, MB (M/LL)	Size, MB (S/LS)	Size, MB (S/LL)
Floating point	1844.7	125.7	1104.8	160.7
IA delta	1118.7	74.8	659.2	92.4
IR delta (W)	1013.8	25.8	562.1	45.7
IR predictive (W)	**984.4**	**24.9**	**431.7**	**37.4**

This table shows the comparison of different intermolecular (IR) encoding strategies using waterfall traversing (W) compared to intramolecular (IA) delta compression. The reported size is a total size of coordinates retrieved from datasets containing the multi-model structures (M) and the structures with repeated subunits (S). Both lossless (LL) and lossy (LS) compression methods were analyzed.

The results suggest that intermolecular algorithms can compress 7.2% more for lossless compression and 39.7% for lossy compression on the dataset with multi-models structures with respect to intramolecular *delta* compression. For structures that contain repeated identical subunits, intermolecular compression saves 20.6% for lossless compression and 34.2% for lossy compression. However, the contribution of intermolecular approaches to the lossless compression of the entire PDB is only about 7% with respect to lossless *delta* compression.

Having considered the different combinations listed above, the overall optimal performance regarding compression and simplicity can be obtained by the combination of integer encoding, intramolecular delta encoding, recursive indexing and GZIP compression. Lossy compression allows to obtain 10-fold reduction in size by reducing the precision of atomic coordinates from three decimal places to one.

## Conclusions

We investigated compression approaches for 3D coordinates of macromolecular structures. The coordinates data contain a high level of entropy and are therefore poorly compressed by the general-purpose compression tools. To achieve better compression, we applied bespoke encoding methods to create a more compact representation of the atomic coordinates. The performance of compression methods was evaluated against benchmark data from the PDB. We demonstrated that the intramolecular compression based on the combination of *integer* & *delta* encoding, *recursive indexing* packing and GZIP entropy compression is very efficient for compressing atomic coordinates of macromolecules with lossless and lossy schemes.

Intermolecular compression approaches can attain additional data reduction compared to intramolecular approaches. However, at the scale of the entire PDB archive the contribution of intermolecular compression is not significant, since it is only applicable to a small fraction of the archive. Therefore, the simple intramolecular *delta* encoding is the preferable choice for efficient compression of macromolecular structures.

The compression approaches investigated in this paper are the foundation for the *MacroMolecular Transmission Format* for 3D structures (http://mmtf.rcsb.org). This format allows a compact representation and interactive visualization of the largest macromolecular complexes that are currently in the PDB [24]. By overcoming I/O bottlenecks such as network transfer, reading and parsing, the entire PDB archive can be loaded into memory within minutes, which opens new possibilities for building scalable analytic tools allowing, for example, interactive structural queries.

The simplicity of the selected compression methods allows for the development of lightweight and fast software libraries for de-/compression. While higher compression ratios can be obtained with more complex algorithms, too little is gained to justify the additional burden on implementation and maintenance. In conclusion, we believe that the compression strategies reported in this article offer important building blocks to face the growing size and complexity of the macromolecular 3D structures.
